# CT volumetry for gastric adenocarcinoma: association with lymphovascular invasion and T-stages

**DOI:** 10.18632/oncotarget.23478

**Published:** 2017-12-15

**Authors:** Xiao-Li Chen, Hong Pu, Long-Lin Yin, Jun-Ru Li, Zhen-Lin Li, Guang-Wen Chen, Neng-Yi Hou, Hang Li

**Affiliations:** ^1^ Department of Radiology, Sichuan Cancer Hospital and Institute, Sichuan Cancer Center, School of Medicine, University of Electronic Science and Technology of China, Chengdu, Sichuan, China; ^2^ Department of Radiology, Affiliated Hospital of Medical School, University of Electronic Science and Technology of China, Sichuan Academy of Medical Sciences and Sichuan Provincial People's Hospital, Chengdu, Sichuan, China; ^3^ Department of Out-Patient, West China Hospital of Sichuan University, Chengdu, Sichuan, China; ^4^ Department of Radiology, West China Hospital of Sichuan University, Chengdu, Sichuan, China; ^5^ Department of Gastrointestinal Surgery, Sichuan Academy of Medical Sciences and Sichuan Provincial People's Hospital, Qingyang District, Chengdu, Sichuan, China

**Keywords:** gastric adenocarcinoma, lymphovascular invasion, T-stages, MDCT, gross tumor volume

## Abstract

**Purpose:**

To determine whether gross tumor volume of resectable gastric adenocarcinoma on multidetector computed tomography could predict presence of lymphovascular invasion and T-stages.

**Results:**

Gross tumor volume increased with the lymphovascular invasion (*r* = 0.426, *P* < 0.0001) and T stage (*r* = 0.656, *P* < 0.0001). Univariate analysis showed gross tumor volume could predict lymphovascular invasion (*P* < 0.0001). Multivariate analyses indicated gross tumor volume as an independent risk factor of lymphovascular invasion (*P =* 0.026, odds ratio = 2.284). The Mann-Whitney *U* test showed gross tumor volume could distinguish T2 from T3, T1 from T2–T4a, T1–T2 from T3–T4a and T1–T3 from T4a (*P =* 0.000). In the development cohort, gross tumor volume could predict lymphovascular invasion (cutoff, 15.92 cm^3^; AUC, 0.760), and distinguish T2 from T3 (cutoff, 10.09 cm^3^; AUC, 0.828), T1 from T2-T4a (cutoff, 8.20 cm^3^; AUC, 0.860), T1-T2 from T3-T4a (cutoff, 15.88 cm^3^; AUC, 0.883), and T1-T3 from T4a (cutoff, 21.53 cm^3^; AUC, 0.834). In validation cohort, gross tumor volume could predict presence of lymphovascular invasion (AUC, 0.742), and distinguish T2 from T3 (AUC, 0.861), T1 from T2-T4a (AUC, 0.859), T1–T2 from T3–T4a (AUC, 0.875), and T1–T3 from T4a (AUC, 0.773).

**Materials and Methods:**

360 consecutive patients with gastric adenocarcinoma were retrospectively identified. Gross tumor volume was evaluated on multidetector computed tomography images. Statistical analysis was performed to determine whether gross tumor volume could predict presence of lymphovascular invasion and T-stages. Cutoffs of gross tumor volume were first investigated in 212 patients and then validated in an independent 148 patients using area under the receiver operating characteristic curve (AUC) for predicting lymphovascular invasion and T-stages.

**Conclusions:**

Gross tumor volume of resectable gastric adenocarcinoma at multidetector computed tomography demonstrated capability in predicting lymphovascular invasion and distinguishing T-stages.

## INTRODUCTION

Despite its decreasing incidence in western countries and china, gastric adenocarcinoma is the fifth most common cancers and the third most common cause of cancer-related mortality worldwide [1, 2]. The 5-year survival following R0 (the surgical margin status was negative) resection remains poor (23–49%), especially for patients with locally advanced disease (14–55%) [3, 4]. To reduce mortality, it is necessary to choose an optimal therapeutic approach, and this, in turn, depends on early detection and accurate preoperative staging [5]. T stage, as an important part of TNM staging system, influences management of gastric cancer directly. For instance, patients with early stage gastric adenocarcinoma has been reported to be rarely accompanied by lymph node invasion, and these patients can receive the less-invasive endoscopic procedure such as endoscopic mucosal resection [6, 7]; For advanced gastric adenocarcinoma, neoadjuvant chemotherapy is an emerging option for marginally resectable gastric cancers as it may lead to downsizing or downstaging of the tumor, thus facilitating its complete resection and improving the patient prognosis [8]. For T4 gastric caner, these patients should be considered for staging laparoscopy because there may be the close relationship between T stage and peritoneal seeding. In addition, lymphovascular invasion (LVI) was an independent factor for lymph node metastasis (LNM) and the prognosis of resectable gastric cancer patients [9–11]. The presence of LVI has been shown to be associated with a high recurrence rate and poor prognosis in patients with gastric cancer. The combination of traditional TNM staging with an assessment for LVI could lead to a more accurate indication of the patient’s prognosis [11]. In addition to TNM staging, LVI has been proved a prognostic indicator that will aid in the identification of gastric caner patients with a higher risk for the recurrence including peritoneal seeding, and these patients should be candidates for more extensive adjuvant chemotherapy to reduce recurrence rates [3, 12]. Therefore, accurate assessment of LVI was also important in predicting prognosis and determining the most appropriate treatment planning.

The pre-operative staging of gastric adenocarcinoma has been based on a multimodality approach, such as endoscopic ultrasonography (EUS), computed tomography (CT), magnetic resonance imaging (MRI) and positron emission tomography. CT and EUS are the two main approaches for preoperative staging of gastric adenocarcinoma. However, the accuracy of EUS and CT for T stage is controversial. There are several contradictory reports about EUS diagnostic accuracy in overall T stage varied from 44.9% to 92.1% [13–16]. Moreover, EUS is invasive, of limited use in severely debilitated patients, highly operator dependent and with a field of view restricted to the gastric wall [16]. CT is widely available, non-invasive, can be performed in most patients and does not require specialized operators. The reported accuracy of CT for T-staging of gastric cancer is variable from 60 to 85% [17–20]. Three-dimensional multidetector computed tomography (MDCT) has been reported to improve the accuracy of preoperative local staging of gastric cancer [21]. Nevertheless, these previous studies investigating the accuracy of CT and EUS in preoperative gastric cancer staging were based on the 6th edition of the American Joint Committee on Cancer (AJCC) Cancer Staging Manual. There have been modifications of the T stage definitions including upstaging of subserosal involvement from T2b in the 6th edition to T3 in the 7th edition and serosal involvement from T3 in the 6th edition to T4a in the 7th edition [22]. These modifications of the T stage may result in inconsistent results. Meanwhile, these studies usually focus on the retrospective studies results and lack of independent validation. CT volumetry of gastric cancer has been first reported to correlate with TNM stage and was better than CT staging [23]. The purpose of this retrospective study was to investigate the capabilities of gross tumor volume (GTV) measured on MDCT for predicting the presence of LVI and distinguishing T stages of gastric adenocarcinoma with pathologic findings in large surgical specimens as the reference standard.

## RESULTS

### Interobserver variability of measuring tumor volume

For the first evaluation in the development cohort of 212 cases, the mean GTV was 32.25 cm^3^ ± 29.25 (range, 2.3–189.3 cm^3^). For the repeat measurement, the mean GTV was 30.89 cm^3^ ± 27.43 (range, 2.5–191.6 cm^3^). As for the precision of the CT measurements of GTV, the coefficient of variation (CV) was 5% (range, 1%–14.6%). Therefore, the CV was less than 10% and interobserver variability of GTV was small, and average values of both measurements were regarded as the final GTV. For the both measurements in four patients, the CV exceeded 10%. Therefore, two additional measurements were obtained and an average of the four measurements was used as the final GTV.

### Univariate and multivariate analysis of clinicopathological factors and GTV correlated with LVI

According to the possible factors predicting LVI including the age, gender, anatomical distribution, histologic type, T stage, GTV and lymph node status, the results of univariate analysis are shown in Table 1. According to univariate analysis, histology type, T stage, GTV and lymph node status showed an association with LVI. LVI was present more frequently in patients with undifferentiated than differentiated (*P* = 0.016), in patients with deeper tumor depth than decreasing tumor depth (*P* < 0.0001), in patient with GTV ≥ 14.5 cm^3^ than <14.5 cm^3^ (*P* < 0.0001), and in patients with LNM than without these involvement (*P* < 0.0001). However, there were no significant associations between the LVI and age (*P* = 0.289), gender (*P* = 0.641), anatomical distribution (*P* = 0.399).

**Table 1:** Univariate analysis of clinicopathological factors and gross tumor volume correlated with lymphovascular invasion

Variables	Lymphovascular invasion		*p* value
	Positive (*n =* 87)	Negative (*n =* 125)	
Age^*^	59.85 ± 11.62	58.8 ± 9.93	0.289
Gender			0.641
Male	62 (71.2)	85 (68)	
Female	25 (28.8)	40 (32)	
Anatomical distribution			0.399
Upper 1/3	23 (26.4)	35 (28)	
Middle 1/3	28 (22.4)	26 (20.8)	
Lower 1/3	36 (51.2)	64 (51.2)	
Histology type			0.016
Differentiated	21 (31)	50 (40)	
Undifferentiated	66 (69)	75 (60)	
T- category			< 0.0001
T1	1 (1.1)	18 (14.4)	
T2	2 (2.3)	38 (30.4)	
T3	10 (11.5)	15 (12)	
T4a	74 (85.1)	54 (43.2)	
Gross tumor volume (cm^3^)			< 0.0001
< 14.5	18 (20.7)	73 (58.4)	
≥ 14.5	69 (79.3)	52 (41.6)	
Lymph node metastasis			< 0.0001
Absent	6 (6.9)	66 (52.8)	
Present	81 (93.1)	59 (47.2)	

Note: Numbers in the brackets are percentages. ^*^Data are medians ± standard deviations.

As regards to multivariate analysis, T stage, GTV and LNM were found to be independent risk factors related to LVI. GTV [*P* = 0.02, odds ratio (OR) = 2.284], T stage (*P* = 0.002, OR = 3.392) and LNM (*P* = 0.000, OR = 11.948) of the primary tumor were associated with LVI.

### Correlation between the LVI and GTV and between T stages and GTV

**Figure 1:**
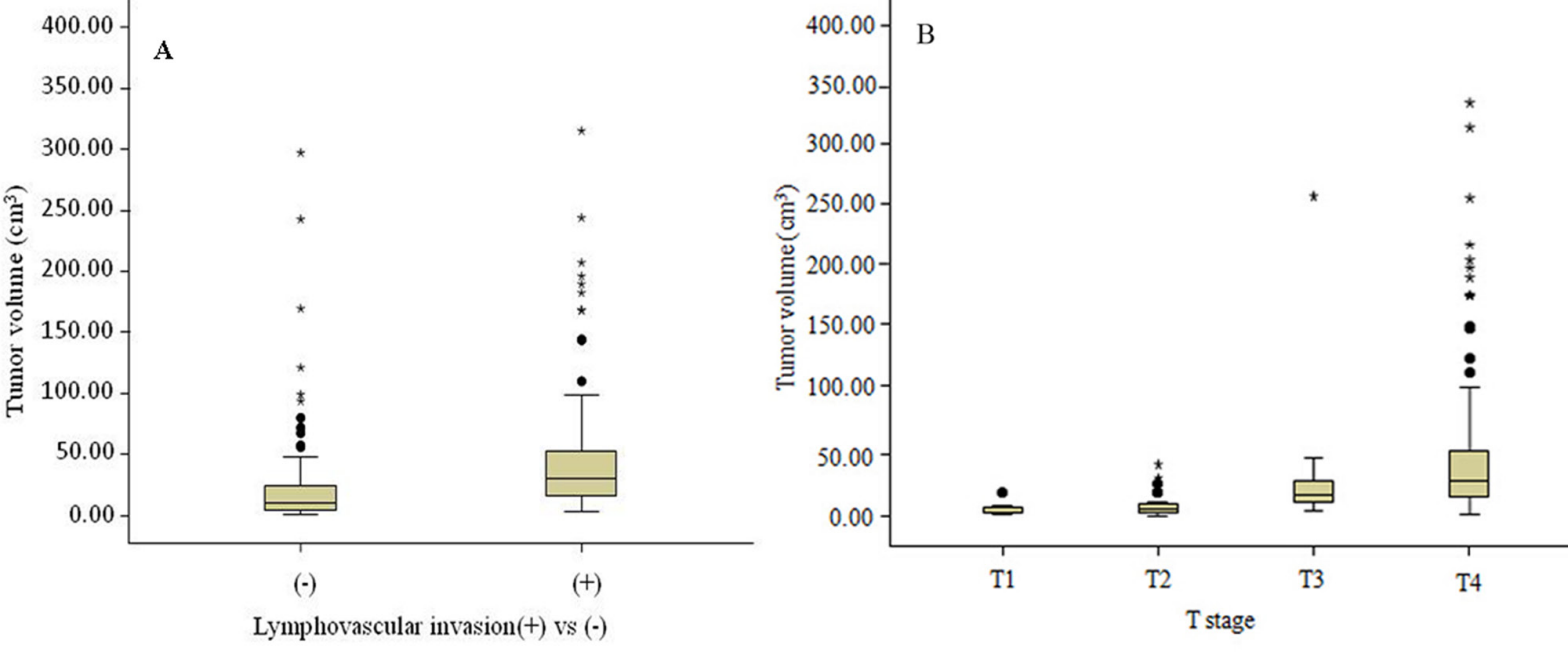
Box plots show the correlation between the lymphovascular invasion and gross tumor volume (GTV) (**A**), and distributions of GTV stratified by T stage of gastric adenocarcinoma (**B**).

GTV increased with the presence of LVI *r* = 0.426, *P* < 0.0001) and increasing of T stage (*r* = 0.656, *P* < 0.0001). The correlation between the LVI and GTV is shown in Figure 1. GTV could predict the presence of LVI (*P* < 0.0001). Table 2 and Figure1 summarize the correlation between T stages and GTV. GTV could help distinguish T2 from T3 stage (*P* < 0.0001), T1-T2 from T3-T4a (*P* < 0.0001), T1 from T2-T4a (*P* < 0.0001), and T1-T3 from T4a stages (*P* < 0.0001). GTV could not help distinguish T1 from T2 (*P* = 0.117) and T3 from T4a (*P* = 0.100).

**Table 2:** Gross tumor volume of resectable gastric adenocarcinoma in patients stratified by T stages in the development cohort

T stages	GTV (*n* =212)
T1	3.24 (2.00, 6.90)
T2	4.90 (3.24, 8.99)
T3	22.21 (11.01, 36.40)
T4a	27 (15.20, 55.34)
T1-T2	4.90 (3.00, 8.00)
T2-T4a	22.52 (8.40, 45.00)
T3-T4a	32 (15.00, 54.00)
T1-T3	8.66 (3.18, 13.02)

Note: Data are presented as median (25th, 75th percentile).

### Receiver-operating characteristic (ROC) analyses of GTV of gastric adenocarcinoma for predicting the presence of LVI and differentiation of T stages

**Figure 2:**
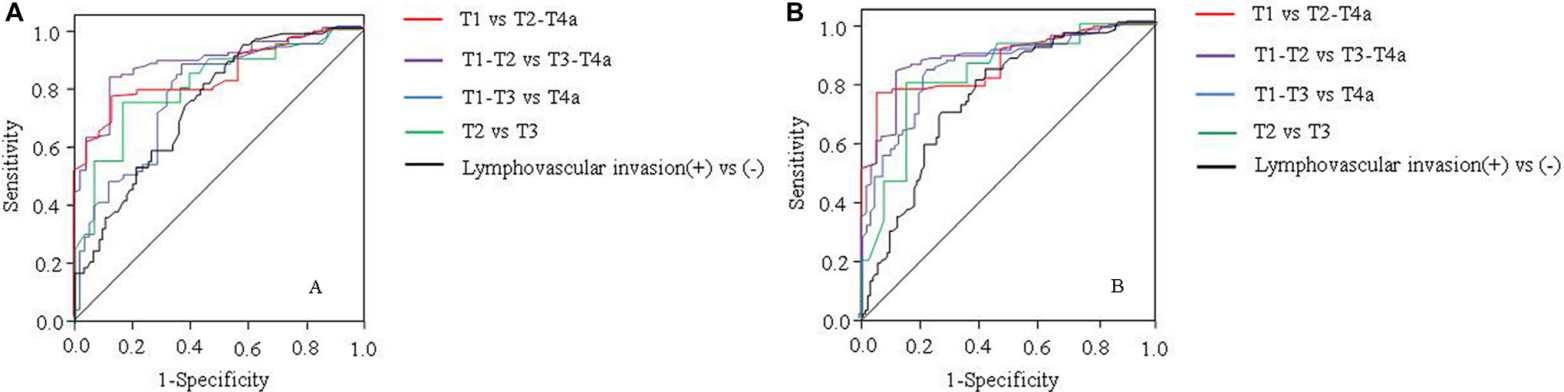
Receiver operating characteristic (ROC) curves of gross tumor volume (GTV) for predicting presence of lymphovascular invasion and differentiation of T stages in patients with resectable gastric adenocarcinoma in the development cohort (**A**) and in the validation cohort (**B**). ROC curves show GTV could help predict presence of lymphovascular invasion, and differentiate T stages between T2 and T3, T1 and T2-T4a, T1-T2 and T3-T4a, and T1-T3 and T4a by using the GTV cutoff value of 15.90 cm^3^, 10.09 cm^3^ and 8.20 cm^3^, 15.88 cm^3^, 21.55 cm^3^, respectively.

As illustrated in Table 3 and Figure 2 in the development cohort, moderate area under the receiver operating characteristic curve (AUC) was observed for GTV in the identifcation of LVI (AUC = 0.76) and higher AUCs for distinguishing T2 from T3 stage (AUC = 0.833), T1 from T2-T4a stage (AUC = 0.860), T1-T2 from T3-T4a stage (AUC = 0.883) and T1-T3 from T4a stage (AUC = 0.834). The GTV cutoff value in identifying LVI was 15.92 cm^3^, with accuracies of 68.8%. For distinguishing T2 from T3 stage, the GTV cutoff value was 10.09 cm^3^, with accuracy of 83.6%. For distinguishing T1 from T2-T4a stage, the GTV cutoff value was 8.2 cm^3^, with accuracies of 80.7%. For distinguishing T1-T2 from T3-T4a stage, the GTV cutoff value was 15.88 cm^3^, with accuracies of 85.1%. For distinguishing T1-T3 from T4a stage, the GTV cutoff value was 21.55 cm^3^, with accuracies of 81.2%.

**Table 3:** Receiver-operating characteristic analysis (ROC) of gross tumor volume of resectable gastric adenocarcinoma for predicting lymphovascular invasion and detecting T stages in the development cohort

Gross tumor volume cutoff (cm^3^)	T stages comparisons	AUC	Sensitivity (%)	Specificity (%)	PPV (%)	NPV (%)	Accuracy (%)
15.92	lymphovascular invasion (+) vs (−)	0.760	75.6	65	59	79.3	68.8
10.09	T2 vs T3	0.833	80	85	66.7	91.8	83.6
8.20	T1 vs T2-T4a	0.860	76	95	99.2	30	80.7
15.88	T1-T2 vs T3-T4a	0.883	83.9	88.1	94.5	69.3	85.1
21.55	T1-T3vs T4a	0.834	83.6	77	86.2	73.1	81.2

Note: AUC = area under the ROC curve, PPV = positive predictive value, NPV = negative predictive value.

As illustrated in Table 4 and Figure 2 in the validation cohort, when compared with data in the development cohort, similar diagnostic performances of GTV were found in the validation cohort in the identifcation of LVI (AUC = 0.742) and distinguishing T2 from T3 stage (AUC = 0.861), identifying distinguishing T1 from T2-T4a (AUC = 0.859), distinguishing T1-T2 from T3-T4a stage (AUC = 0.875), and distinguishing T1-T3 from T4a stage (AUC = 0.773).

**Table 4:** Receiver-operating characteristic analysis (ROC) of gross tumor volume of resectable gastric adenocarcinoma for predicting lymphovascular invasion and detecting T stages in the validation cohort

Gross tumor volume cutoff (cm^3^)	T stages comparisons	AUC	Sensitivity (%)	Specificiy (%)	PPV (%)	NPV (%)	Accuracy (%)
15.92	lymphovascular invasion (+) vs (−)	0.742	73.6	62.7	52	80.5	66
10.09	T2 vs T3	0.861	77.8	77.9	76.4	96.1	88.4
8.20	T1 vs T2-T4a	0.859	73.5	88.9	100	30	76.2
15.88	T1-T2 vs T3-T4a	0.875	81.1	86.4	95.5	67.2	84.3
21.55	T1-T3vs T4a	0.773	81.8	72	80.4	70	76.2

Note: AUC = area under the ROC curve, PPV = positive predictive value, NPV = negative predictive value.

## DISCUSSION

Accurate assessment of LVI and precise staging was important in predicting prognosis and determining the most appropriate treatment planning for patients with gastric cancer. Our results showed GTV could help identify LVI (AUC, 0.760; accuracy, 68.8%), and distinguish T2 vs T3 stage (AUC, 0.833; accuracy, 83.6%), T1 vs T2-T4a stage (AUC, 0.860; accuracy, 80.7%), T1-T2 vs T3-T4a stage (AUC, 0.0.883; accuracy, 85.1%), and T1-T3 vs T4a stage (AUC, 0.834; accuracy, 81.2%) with the cutoff of 15.92 cm^3^, 10.09 cm^3^, 8.20 cm^3^, 15.88 cm^3^ and 21.55 cm^3^, respectively. These results suggest that GTV measured on MDCT can be a potential alternative method for preoperative identifying LVI and distinguishing T stages of gastric cancers.

LVI is not only an independent influencing factor for LNM, but also an independent predictive factor for the prognosis of patients [9, 10]. Previous studies showed the 5-year survival was 76.1% in patients without LVI, while the 5-year survival rate fell to 49.1% in patients with LVI [10]. In addition, the other published studies also have investigated the prognostic significance of LVI in relation to gastric cancer. Peritoneal carcinomatosis has previously been found to be the most prevalent form of cancer recurrence, suggesting that in addition to free cancer cells, hematogenous and lymphatic spread of cancer cells could simultaneously contribute to the recurrence pattern in gastric cancer. The results of these studies indicated that the presence of LVI, either in the blood or lymphatics, correlated with tumor recurrence including peritoneal seeding and a low survival rate that appeared to be independent of lymph node status [12, 24–26]. The addition of LVI assessment to the current AJCC TNM staging system may lead to a more accurate risk stratification of affected patients and may lead to more appropriate clinical decision-making including adjuvant chemotherapy [11, 12]. These results suggest that radical gastrectomy treatment should be combined with appropriate adjuvant therapy to improve their survival. Therefore, the preoperative identifying LVI was important to determine an appropriate treatment plan. In this study, we found that GTV increased with the presence of LVI, and was not only correlated with LVI but also an independent risk factor to LVI. When 15.92 cm^3^ was taken as GTV cutoff value, we had a moderate diagnostic accuracy and AUC for identifying LVI. Until now, few studies use the GTV for predicting LVI. Our findings suggest that GTV on MDCT can be a potential method for the preoperative identifying LVI of gastric cancer. The probably pathological mechanism could be that LVI was mainly observed in the submucosal layer and increase in tumor volume was generally associated with extensive into the submucosal layer by cancer nests [10].

With the new minimally invasive endoscopic mucosal resection therapeutic options for gastric cancer, preoperative differentiation between early gastric cancer (T1) and advanced gastric cancers (T2 or greater) is becoming even more important [5, 6] Our study indicated that we had a higher diagnostic accuracy of 80.7% and AUC of 0.860 using GTV cutoff 8.2 cm^3^ for differentiating T2-T4a from T1 stage. EUS is particularly shown to be useful in predicting T1 stage with accuracy varying between 44.9% and 92.1% [13–15, 27]. These varied accuracies obtained on EUS may attribute to the highly operator dependent and a field of view restricted to the gastric wall. In addition, Ahn et al. reported that the overall accuracy for T1 stage with stomach protocol CT were 86.4% [27]. However, most of these studies had used AJCC 6th edition criteria and had a higher proportion of T1 and T2 stage tumors. Kim et al. evaluated the accuracy of CT gastrography according to the AJCC 7th edition criteria for determining the depth of mural invasion and found that the accuracy for T1 stage was 91% [17]. But the overall diagnostic accuracy of the T staging was 77.2%–82.7%. In their study, population had heterogeneous repartition among different T stage with more than 50% of patients having a T1 lesion. Our population mainly consisted of T4a and have small sample of T1–T3 stage tumors, which may also explain the differences in accuracy of our study as compared to those reported in the literature.

In addition, conventional MDCT determines whether the gastric subserosa (T3) is invaded mainly by a smooth outer margin of the outer layer in the perigastric fat plane. However, the problem in the differentiation between T2 and T3 cancer on CT is that the enhanced low-density-stripe layer can be seen in the inflammatory reaction [28]. We had a higher diagnostic accuracy of 83.6% and AUC of 0.833 using GTV cutoff 10.09 cm^3^ for differentiating T3 form T2 stage. Our findings suggest that GTV can help differentiate T3 from T2 stage. In order to identify patients who best benefit from surgery without preoperative radio-chemotherapy, it was important to distinguish early-to-intermediate (T1-T2 stage) versus advanced (T3–T4 stage, which was likely to benefit from neoadjuvant preoperative chemotherapy) gastric cancer. Joo et al reported that MRI with diffusion-weighted imaging (DWI), MRI without DWI, and MDCT demonstrated diagnostic accuracies of 78.7% to 85.1% [29]. Our study indicated that we had a higher diagnostic accuracy of 85.1% and AUC of 0.860 (GTV cutoff, 15.88 cm^3^) for differentiating T3–T4 from T1–T2. Considering the close relationship between T stage and peritoneal seeding, patients with suspected T4a stage cancers should be considered for staging laparoscopy [30]. Joo et al also investigated that MRI with Diffusion-weighted MRI (DWI), MRI without DWI, and MDCT demonstrated diagnostic accuracies of 72.3% to 76.6% for identifying T4a [29]. Yang et al investigated that the accuracy of conventional CT and iodine concentration was 68.5% and 79.7%, respectively [31]. For differentiating T4a from T1-T3 stage, we had a higher diagnostic accuracy of 81.2% and AUC of 0.834 (GTV cutoff, 21.55 cm^3^). As the serosal surface is very rough and the adjacent adipose tissues are generally turbid, increased density could reflect several different phenomenon including tumor invasion and reactive fibrous connective tissue hyperplasia. Therefore, the accuracy of conventional MDCT is relatively low for identifying T4a stage tumor.

Previous studies have reported that GTV has been shown to correlate with T stage in other cancers, such as oesophageal squamous cell carcinomas and nasopharyngeal carcinoma [32, 33]. As regard gastric cancer, multivariate analysis showed GTV as a significant prognostic factor compared to depth of tumor invasion and nodal status. Hallinan et al. first reported GTV on CT could help differentiate between T stages of gastric cancer [23]. Our study was consistent with that report. However, the cutoff value and accuracy obtained in that study was a little higher than ours. The probably reason was that a predominance of T4 stage in that study may consist of T4a and T4b, which is different from our study consisting of only T4a stage. It was known to all that most patients with T4b stage tumor had large tumor volume. Moreover, a limited number of T3 stage were included. Therefore, additional studies with a subsequently reduced selection bias, and including more T1–T3 stage tumors, will be needed. Despite all this, it should be noted that these published previous studies did not apply the cutoff values identified in the development cohort to a completely independent validation cohort and compare the preoperative T stages to the postsurgical staging and then calculate the accuracy of recommended CT methodology. In our study, high diagnostic accuracy of GTV in differentiating between T stages was found, with AUCs of 0.760–0.883 in the development cohort and 0.742–0.875 in the validation cohort.

There were several limitations in our study. Firstly, the patient numbers among different T stage were not well balanced. We had more patients with T4a and less patients with T1 and T3 stage than other studies. This could be partly attributed to inadequate early gastric cancer screening programs in our country. Meanwhile, our study population excluded patients with the T4b stage tumor. We believe that MDCT is the method of choice to image stage T4 tumors because surrounding organs invasion are clearly shown in relation to the gastric tumor. Therefore, it was not essential to utilize GTV to predict the T4b stage. Secondly, our study did not include lymph node staging. However, previous study has demonstrated that GTV could help differentiate between N stages in gastric adenocarcinoma [34]. Finally, GTV measurement can be time consuming with the method we described. Advanced measurement software may significantly reduce the time needed to compute the tumor volume. In our centre, the time for well trained radiologists performing the measurements and calculation of GTV was controlled in 200 sec. Moreover, we have examined approximately 5 case of newly diagnosed gastric carcinoma per week and volumetry did not have a significant impact on workflow.

In conclusion, GTV of resectable gastric adenocarcinoma measured with MDCT is associated with LVI and the T stages. GTV has higher diagnostic accuracy in indentifying LVI and differentiating between T stages in gastric adenocarcinoma. We believe that this study could be helpful in quantitatively predicting the LVI and differentiating between T stages for clinicians choosing optimal treatment modalities for individual cases.

## MATERIALS AND METHODS

### Study patients

This retrospective study was approved by our Ethics Committee, with a requirement for written informed consent. Between June 2013 and July 2016, 450 consecutive patients (age range, 20–81 years) with gastric adenocarcinoma diagnosed in our institution were retrospectively recruited into this study. The exclusion criteria for this study were as follows: (a) 17 patients who had contraindications to surgery (including 3 patients with other major organ severe disease, 8 patients with haematogenous metastasis, and 6 patients with direct invasion of adjacent organ did not undergo operation), (b) 28 patients who had been treated with preoperative neoadjuvant chemotherapy or radiation therapy, (c) 5 patients whose tumor images were of poor quality, (d) 40 patients who had T4b stage. Consequently, this study involved 360 patients (age range, 22–79 years). Then these patients were divided into two cohorts. In the development cohort (group A), data in 212 patients were used to develop the GTV cutoff values in identifying the presence of LVI and differentiating T stages of gastric adenocarcinoma. In the validation cohort (group B), data in 148 patients were used to validate the developed GTV cutoff values.

All patients underwent preoperative contrast-enhanced CT examinations and endoscopic biopsy. Subsequently, the enrolled patients were scheduled for standard operative procedures. The interval between CT and surgery was less than one week. As regards to the group A, according to the postoperative pathologic examination, 87 patients had LVI while 125 patients did not. LVI was defined as the invasion of vessel walls by tumor cells and/or the presence of tumor emboli within an endothelial-lined space; with no distinction between vascular and lymphatic vessels [35]. The following criterion was used to identify the lumen of blood and/or lymph vessels: (1) lined by endothelium; (2) with supporting smooth muscle or elastica; (3) filled with lymphatic fluid or red blood cells. The tumors were located in the upper one-third of the stomach in 58 patients, the middle one-third in 54 patients and the lower one-third in 100 patients. Tumor histology was classified into two groups according to the Lauren classification [36]: the differentiated group [well- or moderately differentiated adenocarcinoma] in 71 patients, and the undifferentiated group [poorly or undifferentiated adenocarcinoma] in 141 patients. According to the postoperative histopathology and 7th edition AJCC criteria [22], primary tumors were classified as T1 stage in 19, T2 stage in 40, T3 category in 25, and T4a stage in 128 patients. As regards to the group B, 54 patients had LVI while 94 patients did not. Primary tumors were classified as T1 stage in 15, T2 stage in 28, T3 stage in 16, and T4a stage in 89 patients.

### Contrast-enhanced MDCT

Five minutes prior to the CT examination, all patients received 10 mg of butylscopolamine bromide (Buscopan; Boehringer Ingelheim, Ingelheim, Germany) to minimize the peristaltic bowel movement. Patients received with gas ingested effervescent granules with 500 mL of water to distend the stomach. Patients were examined in the supine position, and the CT data acquisitions were obtained in the arterial phase (25–30 s) and portal-venous phase (60–70 s) covering the entire stomach in arterial phase and entire abdomen and pelvis after initiation of the contrast material injection (Ultravist 300, Iopamidol; Bayer Healthcare, Berlin, Germany). Examinations were performed during one breath hold at full suspended inspiration. The CT scanning variables were 120 kVp, 200–380 mA, section thickness of 2 mm, and reconstruction interval of 2 mm. Scanning was performed during the arterial and portal venous phases, and the anatomic coverage was from the apex of the lungs to the pelvic cavity. The data were directly interfaced and forwarded to the General Electric Advantage Workstation 4.4 (Advantage Workstation version 4.4; General Electric Healthcare).

### GTV measurement

**Figure 3:**
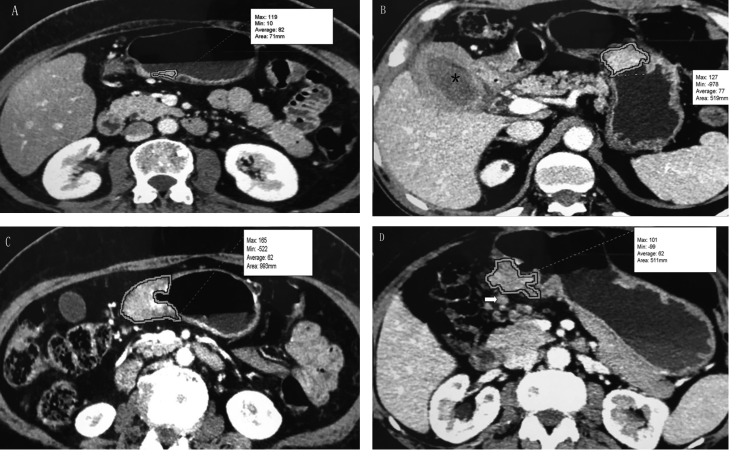
Tumor volume measurement on MDCT (**A**) T1 stage gastric adenocarcinoma on the gastric angle in a 58-year-old man. Tumor area is manually drawn along margin of tumor, and value of this area (71 mm) is automatically derived by software together with minimal, maximal, and average CT attenuation (in Hounsfeld units). (**B**) T2 stage gastric adenocarcinoma on the gastric body and xanthogranulomatous cholecystitis (^*^) in a 55-year-old man. The tumor had an area of 519 mm. (**C**) T3 stage gastric adenocarcinoma on the gastric antrum in a 48-year-old man. The tumor had an area of 993 mm. (**D**) T4a stage gastric adenocarcinoma on the gastric antrum in a 67-year-old man. The tumor had an area of 511 mm. Lymph node metastasis was found adjacent to the tumor (arrow).

GTV was measured at a window width of 380 HU and window level of 50 HU. The portal venous phase was used for GTV measurement. GTV was calculated by multiplying the sum of all the tumor areas by the section thickness according to a previous report [23, 34, 37]. For delineation of tumor area, we regarded the gastric wall as abnormal when its thickness was ≥ 5 mm on transverse imaging with the stomach distended [19]. Tumor area was manually outlined on each axial enhanced CT image (Figure 3). Sometimes the accurate manually delineation of tumor area is difficult because the tumor may locate on oblique plane on axial CT images. Therefore, coronal or sagittal reconstruction images were obtained on the General Electric Advantage Workstation. The reviewers were given the option to use coronal or sagittal reconstructed images to determine the tumor area when the tumor may locate on oblique plane on axial CT images.

Tumor areas were automatically derived by the software. This previous process and analysis were repeated for each contiguous transverse level until the entire tumor had been covered, and the values of each contiguous transverse level were then summed to calculate the GTV. The time required to perform the measurements and calculation of GTV was approximately 200 seconds on average (range, 100 to 350 seconds).

To maintain the accuracy of the measurement, 2 experienced radiologists (a 4 year radiology fellow and an attending radiologist with 10 years of specialisation in abdominal imaging) who were blinded to all clinical and pathologic data including endoscopic findings working in consensus were trained in measuring the GTV randomly in another 20 patients by a radiologic professor. All tumor measurements were repeated one month later to test the interobserver reproducibility of the measurement of GTV.

### Statistical analysis

All statistical analyses were carried out with SPSS (version 17.0, SPSS, Chicago IL, United States). A *P* < 0.05 was considered to represent a significant difference. The CT data of the 212 patients with gastric adenocarcinoma were used to test interobserver reproducibility of the measurements. In these 212 patients, the precision of the replicated GTV measurements was assessed using CV (standard deviation / mean × 100). When the % CV was less than 10%, interobserver variability was considered to be small, and the averaged value of the two observers’ measurements was regarded as the final GTV. If the % CV exceeded 10%, another two measurements were made by the previous observers and an average of the four measurements was used as the final GTV.

Univariate associations between LVI and GTV and clinicopathological factors were analyzed using the chi-square test (or Fisher’s exact test when appropriate). Multivariate logistic regression analyses were used to assess the associated risk factors for LVI. GTV were compared between patients stratified by T stages using non-parametric Mann-Whitney tests together with Bonferroni correction for multicomparisons. If there were significant positive findings on Mann-Whitney tests, the cutoff values of GTV were then determined with ROC analysis for predicting presence of LVI and differentiation of T stages. For the identification of T stages of gastric cancer, accuracy, sensitivity, specifcity, positive predictive value, and negative predictive value were calculated with an optimal cutoff value that maximized the sum of sensitivity and specificity.

## Abbreviations

GTV: gross tumor volume
LVI: lymphovascular invasion
AUC: area under the receiver operating characteristic curve
EUS: endoscopic ultrasonography
AJCC: American Joint Committee on Cancer
CV: coefficient of variation
DWI: diffusion-weighted imaging
ROC: receiver-operating characteristic
MRI: magnetic resonance imaging
DWI: diffusion-weighted MRI
MDCT: multidetector computed tomography
CT: computed tomography
